# Health Workers in Sub‐Saharan Africa: Concurrent Skilled Health Worker Shortages and Under‐Employment

**DOI:** 10.1002/hpm.70001

**Published:** 2025-06-11

**Authors:** Pieternella Pieterse

**Affiliations:** ^1^ Institute of Global Surgery Royal College of Surgeons in Ireland Dublin Ireland

**Keywords:** bilateral agreements on health worker migration, health worker migration, human resources for health, world health organization health workforce support and safeguards list

## Abstract

In 2021, the World Health Organization (WHO) introduced the Health Workforce Support and Safeguards List, updating the 2010 WHO Global Code of Practice on the International Recruitment of Health Personnel. The change introduced a new way of defining what constitutes a country with a critical health worker shortage. The new calculations are based on a combined score of countries' health worker density per 1000 population and the Universal Health Coverage (UHC) service coverage index. It has led to an increase in the number of low‐ and middle‐income countries (LMICs) considered at risk from active recruitment by high income countries (HICs). However, the 2021 WHO Safeguard list review failed to explicitly recognise the main causes of low health worker density in countries on the list. Many included countries are unable or unwilling to invest in their health sectors, which restricts the number of staff that can be hired. These countries experience high unemployment among trained and qualified health workers, despite their high need for health workers. Recent dramatic reductions in international aid and development support, means that LMICs that fail to invest in their health workforce, will face ever greater shortfalls in meeting the basic health needs of their populations. For WHO Safeguard‐listed countries establishing bilateral health worker migration agreements, better support is needed to create fair deals that allow them to receive compensation from destination countries for the training costs of their emigrating health workers, which can be used to directly hire additional health workers back home.

**Summary**

- Insufficient funds to hire health workers means many LICs in Africa have unemployed health workers, despite struggling with low health worker density
- The 2023 WHO Safeguard list includes 37 African countries, out of 55 in total
- Past bilateral health worker migration agreements have not always provided tangible benefits to countries on the WHO Safeguard list
- Better bilateral agreements can benefit LICs with paradoxical health worker (HW) surpluses by channelling funds to HW recruitment

## 1 Health Worker Shortages and the WHO Global Code

Health workers of all kinds—medical doctors, nurses, midwives, allied and auxiliary cadre workers, pharmacists and laboratory staff—are central to health systems. How many and what type of health workers are needed for an optimally functioning health system has been debated for many decades [1, 2]. The conclusion is that it depends on the context, especially: distribution and demography of the population, disease burden, and the facilities and skills mix available. For low‐income countries (LICs) in sub‐Saharan Africa (SSA), the size and composition of the health workforce is being limited by meagre funding allocations, while domestically trained human resources for health are increasingly available [3]. As a result, trained nurses, midwives, and occasionally doctors, are finding themselves unemployed, composing an ever larger ‘paradoxical surplus of health workers’ [4]. This challenge has yet to be widely recognized. Suitable policy responses need to be formulated in order to optimise health workers availability for the world's growing and ageing population.

The World Health Organization (WHO) has, for nearly 2 decades, taken on a relationship brokering role between high‐income countries (HICs) and LICs in relation to health worker mobility [5]. In recent years, HICs have seen their demand for health workers increase, while they are failing to train and graduate sufficient numbers of health workers to meet their needs [6]. LICs have seen a continued outflow of their most experienced and best educated workers, with countries that have low health worker ratio somewhat protected from international recruitment by WHO's instruments [5]. However, in SSA, the health workforce context has evolved. In this *perspective*, the change in supply and demand for health workers in SSA will be examined, in order to highlight why better bilateral health worker migration pacts are needed.

In 2021, WHO reported that, in the preceding decade, the number of migrant doctors and nurses working in Organisation for Economic Cooperation and Development (OECD) countries had risen by 60% [7]. In addition, ‘Education capacity and ability to retain health workers in areas of greatest need remain constrained across countries. Active and unethical recruitment practices remain an important concern for many countries’ [8]. India and the Philippines have long been the main source countries for migrating doctors and nurses, respectively [9, 10]. However, statistics show increasing numbers of migrating health workers grew up and were educated in SSA [11].

African countries have been more vulnerable to the negative impact of health worker migration than other parts of the world, due to their low health worker to population density [12]. Many SSA countries suffered significantly from the impacts of structural adjustment policies imposed in the 1980s, which led to a reduction in their public sector wage bills. In 1998 the average ratio of doctors per 10,000 was 1.71 in SSA, compared to an average of 30.37 in selected industrialized countries; and for nurses, 8.97 per 10,000 population in SSA, compared with 72.36 in industrialized countries [13].

It was in this context that in 2004, the World Health Assembly mandated that a code of practice on the international recruitment of health workers was formulated [5], which led, in 2010, to the adoption of the Global Code of Practice on the International Recruitment of Health Personnel. It set out voluntary principles and practices to guide the ethical international recruitment of health workers. Central is the recommendation that ‘Member States should discourage active recruitment of health personnel from developing countries facing critical shortages of health workers’ [14]. The WHO Global Code was updated in 2021 and is now called the Health Workforce Support and Safeguards List, to reflect a shift in thinking on how ‘critical shortages of health workers’ should be defined [15].

The revised measurements mean that countries are included if unable to achieve a skilled health worker density of 44.6 per 10,000 population and maintain a UHC service coverage index of 50. The number of included countries changed from a total of 57 that were included in the initial 2006 list, to 47 in 2021 and 55 since 2023 (when the UHC threshold was raised to 55). Out of the current 55 countries on the list, 37 are in the WHO African region [5, 16]. While the update changed the critical shortage of health workers definition, the spirit of the list remains the same; to protect the included countries from widespread and unorganised active recruitment of health workers by high‐income, in favour of bilaterally organised migration.

The WHO Global Code did not seek to ban recruitment from resource poor countries on the list, it suggested that recruitment from countries on the WHO Global Code list should only be arranged via bilateral agreements. The intention was, and is, to compel countries that plan to hire health workers from countries with critical shortages, to agree to pay compensation, in recognition for the investment the country‐of‐origin have made to train the emigrating health workers [17].

## 2 Bilateral Health Worker Migration Arrangements Involving WHO Safeguard List Countries

While the strength of the WHO Global Code/Safeguard list lies undoubtedly in the fact that it is widely known, the bilateral agreements that the list has given rise to, have been disappointing. A 2024 study by the WHO and the OECD examined 150 bilateral agreements in order to formulate better guidance on the components that future agreements should contain [18]. Clear guidance was lacking in the past, which may have been deliberate, given the diverging views the UN member states who signed up to the initial treaty [17]. The authors of the 2024 bilateral agreements report highlighted that the ‘…potential of government health worker migration and mobility agreements to strengthen the health systems of countries of origin has yet to be realized, despite it being central to the objectives of the Code’ [18]. The report notes that ministries of health were not involved during a significant number of bilateral agreement negotiations and suggests a lack of negotiation capacity within country‐of‐origin government teams, and, as the report's authors pointed out, socio economic imbalances and power dynamics favour the recipient countries which ‘have little incentive to support health systems in countries of origin’ [18]. As a result, past health worker migration agreements have lacked concrete plans of what countries of origin could expect in return for agreeing to facilitate the outmigration of their health workers. Other studies have also noted that health systems strengthening commitments within these pacts can be vague, and lack agreed monitoring tools, leading to commitments remaining unfulfilled [19]. The disclosure of bilateral agreements to the general public can be delicate. Ghana and the UK's 2022 agreement, whereby a compensation of ‘up to £1000 per nurse’ was reportedly being paid by the UK to Ghana's health system received negative attention in both countries. Accusations of slavery appeared in the media [20], demonstrating its sensitivity [21]. There are also positive examples; the so‐called ‘triple win’ approach to migration, that provide benefits to both the country of origin (by easing oversupplied labour markets), health workers (better wages, sending remittances home) and destination countries (provided much needed healthcare staff) is thought to be based on Germany's bilateral health worker migration agreements [22]. While it too has been criticised [23], it has undeniably raised the standards for good health worker immigration practices; Germany's voluntary ‘fair recruitment certificate’ is not yet ‘industry standard’, but, ‘at least larger recruitment agencies, and some employers, now seek certification’ [23].

## 3 Overlooking Africa's Paradoxical Surplus of Health Workers

The need for improved support to negotiate better bilateral health worker migration pacts for countries on the WHO Safeguard List is more urgent than ever. Less than a decade ago, the first significant changes were witnessed in trendlines in SSA with regards to health worker employment, retention and production [2]. While much of the world faced increasing *needs‐driven* health worker shortages, which meant that countries had funding to recruit staff but a shortage of available health workers, SSA countries were seeing concurrent *demand‐driven* shortages while health worker supplies, of nurses in particular, were increasing. Health workers were needed *and* were being produced in SSA countries, but there was not enough funding in the health sector to employ them [3]. This has resulted in a paradoxical surplus of unemployed health workers [4], despite significant needs‐based health worker shortages and poor health outcomes. This makes SSA a significant outlier in a context where a 10 million shortage of health and care workers is predicted to affect the world by 2030 [24]. Recent studies have suggested that unemployment among health workers in SSA may be as high at 24%, based on data from ten African countries, some of which are on the WHO Safeguard list [25]. The shortage of funding for the health wage bill can have several reasons: Many LICs struggle with good governance, which has been associated with low levels of tax revenue, even in countries where significant natural resources are being extracted and exported, which, theoretically, should boast exchequers and provide ample funding for basic public services [26]. Even when there is sufficient funding available to governments, health and other public services can be less of a priority for ideological reasons or during conflict [27]. Between 2016 and 2021 a total of 13 safeguard countries were requested to decrease public wage bills by 0.2–11% in order to qualify for, or adhere to, IMF loan conditionalities [25].

Studies from Sierra Leone, Liberia, Guinea, Nigeria and Niger [28, 29, 30, 31] have all produced evidence to suggest that many health workers who do not have secure employment in the health sector are employed in an informal manner within the public health system, which is often associated with negative impacts such as the charging of informal fees. These studies also highlight challenges such as higher levels of absenteeism among the official health workforce, who are employed and paid, but absent, being replaced by informal health workers; and managerial staff lack clarity on health worker distribution, which leads to suboptimal planning and supply of health workers.

Having a large unemployed health workforce appears to negatively impact healthcare delivery and health outcomes due to the informal practices that many trained but unemployed health workers are forced to resort to. Most of the unemployed health workers in countries of the WHO Safeguard list are needed in their own countries and would obtain health sector jobs locally if their ministries of health and formal, regulated private healthcare providers had sufficient funding to meet the needs‐based human resources for health requirements [3]. Given their countries' inclusion on the WHO Safeguard list, it means that they are often struggling to find employment, both domestically and internationally.

Consequently, there is a need for better bilateral migration agreements. In an ideal world, no health workers from WHO Safeguard listed countries should feel the need to emigrate abroad for employment, for a better life, especially at a time when their skills are badly needed at home. However, current circumstances are not ideal, far from it. Recent cuts in aid by the United States and several other major donor countries, have left many LIC health systems more under‐resourced than ever. Firstly, it is important that this situation is not exploited by high‐income countries, especially by those that have recently cut or abolished such aid to countries from which they wish to recruit. Secondly, at‐risk LICs need to be supported to get exemptions to loan conditionalities that prevent them from employing their health workers. Thirdly, countries need to be supported by international partners to draft, negotiate and enter into bilateral agreements with recruiting countries to limit recruitment; and/or compensate for the loss of unemployed health workers, where it can afford to lose some health workers to international recruitment, preferably for a temporary period of time.

The 2024 WHO/OECD report evaluating past bilateral agreements, points out some minimum requirements that are not always achieved, but should be fought for: ‘…agreements, when negotiated with participation from health stakeholders and ensuring that the domestic supply of health workers being negotiated for is adequate, should provide necessary investment in countries of origin to improve their health outcomes’ [18]. Not only should bilateral agreements on health worker migration provide countries of origin with reasonable compensation for the training and investment for health workers that are now leaving, the way in which such compensations are received and used, should also be structured in a manner that can genuinely benefit the country‐of‐origin's health system. In other words, if a country has a significant (over)supply of health workers, but the challenge is insufficient funding to recruit them, bilateral funding to train additional health workers may not contribute much to improving its health system; and may in fact be a way of pump‐priming the supply line of health workers for international recruitment. Instead, a bilateral agreement could really optimise its impact if it were possible to channel funds into health worker salaries. This too has been suggested in the WHO/OECD report, stating that ‘measures to increase fiscal space to recruit specific types of health workers should also be considered’ [18].

## 4 Moral Obligations, Reliance on LMIC Health Workers, and Economic Sense

There are many reasons why high‐income countries, which are the predominant destination countries for health workers migrating from low and middle income countries (LMICs), should adopt a more ethical stance regarding health worker migration. There is a moral obligation, considering that SSA carries an estimated 25% of the world's disease burden but accounts for only 4% of the global healthcare workforce [32]. The projected shortage of approximately 10 million health workers worldwide is expected to affect the WHO African and Eastern Mediterranean regions most severely [24]. Aid cuts to LMICs have been suddenly made, with no opportunity for countries to look for substitute funding and with scant regard to any of the lessons that were learnt during COVID‐19, that poor health, infections and outbreaks in one part of the world can affect those in other parts too.

The recent, post‐COVID surge of high‐income country recruitment of health workers suggests that the reliance on LMICs for the supply of doctors and nurses is far from over. A 2023 report shows that 35% of workers from Zambia and 63% of workers from Ghana intended to migrate, mostly to OECD countries; 20% of Zimbabwean born doctors and nurses are already working in the UK [11]. This suggests that SSA countries on the WHO Safeguard list are important sources of health worker supply and deserve to be supported to ensure that health outcomes are not adversely affected by the migratory pull of high‐income countries. This is not only important from an ethical standpoint, it may also make economic sense for destination countries to do so.

## 5 Safeguarding From Exploitation

Nurses and lower cadre health and care workers are predominantly female, which adds a gender dimension to this already complicated migration story, one in which exploitation needs to be guarded against at multiple levels, and for a range of different reasons [33]. High income countries taking advantage of resources being ‘produced’ in the Global South follow a well‐rehearsed pattern of colonial and post‐colonial exploitation [34]. While the data cited in this article, and much of the data regarding health worker migration, focuses on doctors, nurses and midwives, so‐called skilled health workers, there is a significant number of health and care work‐providing people, mainly women, who are less qualified and whose migration patterns are less well documented. Their work in high‐income countries is more likely to be precarious, and they themselves are more likely to suffer from exploitation [35]. In addition, there is the scenario, where doctors, nurses and other qualified professionals, trained to a high standard in their country of origin, face a lack of recognition of their qualifications. They are recruited to work in posts in HICs that do not require their level of training, for example in low‐skilled employment to care for the elderly. This is a waste of a scarce and much needed resource that would be better utilised in LMIC [36].

Migration happens due to economic pull factors, but push factors also need to be recognised. Health worker unemployment in SSA is strongly related to countries' limited fiscal space and the meagre budgets that some governments choose to allocate to health. High levels of debts servicing, international lending institutions' loan conditions and the cost‐of‐living crisis have all affected African countries' limited health budgets. So have wars, internal conflict, and poor governance, which together have contributed to many countries' failure to progress [37]. Yet there again, internal conflicts and wars are usually fuelled, directly or indirectly, by HIC actors and economic interests. The majority of the countries on the WHO Safeguard list have suffered, or continue to suffer, from the challenges described above, yet many also have young populations and governments which have been able to increase health worker training opportunities by expanding public training institutions, or allowing private sector actors to do the same. Large young and educated cohorts of health workers across the continent ought to be signs of hope and optimism.

It is time that bilateral agreements on health worker migration become instruments that can positively contribute to the growth and strength for country‐of‐origin health systems. While in the past, negotiations on health worker migration pacts have favoured HICs and short‐changed the Global South, this does not have to be this way in the future. It is vitally important to understand the challenges that countries on the safeguard list face regarding their health systems, and, as the WHO/OECD report states, support improved bilateral health worker pacts to truly benefit health systems of countries of origin. It is ‘not just an ethical and moral responsibility, it is also in the interest of health workforce sustainability of destination countries, global health security and economic growth’ [18].

## 6 Summary of Action Points

Countries engaging in health workforce recruitment from countries on the WHO Safeguard list should routinely seek to engage in bilateral migration agreements rather than allowing commercial recruitment agencies to arrange such migration informally.

Donor agencies should provide countries on the WHO Safeguard list with technical expertise to negotiate health worker migration agreements effectively, securing the best possible arrangement for the county of origin.

Compensations to WHO Safeguard listed countries should be designed in such a way that funds contribute to alleviating some of the causes of health worker shortages.

International lenders should consider exemptions to loan conditionalities that prevent LICs with poor health worker ratios from employing their health workers.

WHO Safeguard listed countries should be supported, but also held accountable in international fora, to ensure they improve their tax revenue collection, from extractive industries especially, and budget adequately for basic public services such as health.

Gender equity and ethical recruitment practices should be central to all health worker migration agreements by clearly documenting workers' rights, professional recognition, working conditions, remuneration, and access to health and care services for migrating staff.

## Ethics Statement

The author has nothing to report.

## Conflicts of Interest

The author declares no conflicts of interest.

## Data Availability

The author has nothing to report.
